# Engineered Strain in 2D Materials by Direct Growth on Deterministically Patterned Grayscale Topographies

**DOI:** 10.1002/advs.202522850

**Published:** 2026-02-10

**Authors:** Berke Erbas, Arindam Bala, Hernan Furci, Anushree Dutta, Naresh Kumar, Renato Zenobi, Giovanni Boero, Andras Kis, Juergen Brugger

**Affiliations:** ^1^ Microsystems Laboratory École Polytechnique Fédérale de Lausanne (EPFL) Lausanne 1015 Switzerland; ^2^ Laboratory of Nanoscale Electronics and Structures École Polytechnique Fédérale de Lausanne (EPFL) Lausanne 1015 Switzerland; ^3^ Department of Chemistry and Applied Biosciences ETH Zurich Zurich 8093 Switzerland

**Keywords:** 2D materials, grayscale nanopatterning, strain engineering, strain‐engineered 2D material growth

## Abstract

Strain is a proven technique for modifying the bandgap and enhancing carrier mobility in 2D materials. Most current strain engineering techniques rely on the post‐growth transfer of these atomically thin materials from growth substrates to target surfaces, limiting their integration into nanoelectronics. Here, we present a new approach where strain in 2D materials is already introduced directly *during* their growth on grayscale‐patterned topographies instead of flat surfaces. Both strain levels and orientations are deterministically engineered by controlling grayscale surface contour lengths through thermal expansion mismatches in nanostructured stacks, where the conformally grown and firmly attached 2D material is forced to match the underlying morphology change during cooling. With this method, we experimentally demonstrate precise control of localized tensile strain from 0 to 0.5% in grown MoS_2_ monolayer along both uni‐ and multiaxial directions, while higher strain levels are shown to be theoretically possible. This strain‐engineered growth of 2D material films directly on the target substrates is a generic and adaptable approach to various combinations of grayscale‐thin‐film/substrates and eliminates all the transfer‐related limitations of previous approaches, thus paving the way for integrating strained 2D materials into next‐generation nanoelectronics.

## Graphical abstract

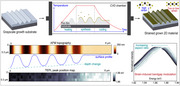

## 1 Introduction

Semiconductor device innovations have historically relied on dimensional downscaling [1], but as lithographic advancements approach their physical limits, innovations now focus on new device architectures and materials for *More Moore* [2, 3, 4]. Beyond dimensional scaling, device functionality is being added or enhanced to enable novel applications with a *More‐than‐Moore* vision [2, 5]. The introduction of the third dimension at the device level, which started with fin field‐effect transistors (FinFETs) and, more recently, has continued with gate‐all‐around field‐effect transistors (GAAFETs), has been a pivotal milestone in architectural innovation and a forefront example [6, 7], a trajectory that is expected to continue with 3D stacking for next‐generation computing hardware [8]. In terms of materials, replacing or complementing silicon channels with 2D semiconductors, particularly transition metal dichalcogenides (TMDs) such as molybdenum disulfide (MoS_2_), shows promise due to their sub‐nm thickness for increased transistor density and low off‐currents for energy efficiency [9]. However, beyond‐silicon technology requires further advancement through research to develop nanoelectronics‐compatible integration solutions and to outperform the state‐of‐the‐art performance of silicon transistors while continuing dimensional scaling.

2D material integration typically involves polymer‐assisted material transfer, including detachment, alignment, and transfer of atomically thin materials from the growth substrate to the target device. While most research focuses on improving growth quality by reducing intrinsic defects, the transfer process undermines these improvements with extrinsic defects and contamination [10, 11]. Thus, high‐yield integration is constrained by the transfer process. To enable the shift from today's silicon devices to tomorrow's 2DM‐based electronics, higher‐quality 2DM growth directly on target substrates has recently been considerably studied [12, 13]. Additionally, current 2D transistors based on synthesized 2D materials such as MoS_2_, as the most prominent example, commonly exhibit electron mobilities of <30 cm^2^/V·s [12, 14, 15, 16, 17], due to electron‐phonon scattering [18], while high‐performance transistors typically require mobilities of ≥100 cm^2^/V·s [4]. Strain engineering, a performance‐enhancing technique used in commercial silicon complementary metal–oxide–semiconductor (CMOS) technology since the *90 nm node* [19], now holds similar promise for 2D materials.

Strain in silicon transistors improves FET mobilities by lowering scattering through a modified silicon band structure, which is typically achieved through lattice mismatch with silicon–germanium or by using stressed thin‐film nitride capping [20]. Similarly, strain in 2D materials, including graphene [21], but particularly 2D semiconductors such as molybdenum disulfide [22], tungsten disulfide [23], and tungsten diselenide [24], offer bandgap engineering potential and have demonstrated enhancements in electron mobility by modifying the band structure to reduce electron–phonon scattering [18]. However, achieving industry‐compatible integration of these materials remains a difficult engineering challenge. Various methods, including displacement via microelectromechanical systems [21], direct mechanical or thermomechanical nanoindentation [25, 26], substrate heating for expansion [27], flexible substrate bending or stretching [22, 28, 29, 30, 31], and pressurized bulging [32], have been used to show the effects of strain in 2D materials. These approaches are primarily limited to fundamental studies involving external actuation through pressure, heat, or force and are not readily compatible with large‐scale industrial integration.

After fundamental knowledge was developed on the effects of strain on the optical and electrical properties of 2D materials, recent research has shifted to CMOS‐compatible and scalable strain engineering strategies, including thermal expansion mismatch [24], thin‐film stressor capping [23, 33, 34, 35], and straining on pre‐structured substrates [36, 37, 38, 39, 40, 41, 42, 43]. These approaches all have trade‐offs between strain magnitude, substrate compatibility, and fabrication viability. Thermal expansion mismatch between the substrate and the grown 2D material [24] has been used to induce strain during the growth, but this approach prioritizes the thermal properties of the substrates over the application requirements, such as the use of silica to obtain a reasonable tensile strain in 2D material like WSe_2_. Additionally, in this approach, the level of strain is predetermined by thermal expansion mismatches, and varying strain levels and orientations on the substrate cannot be achieved, as the same strain is globally and homogeneously distributed in all directions. Inspired by the SiN stressor capping used in strained silicon technology [20], an improvement in on‐current of MoS_2_ FETs using a SiN_*x*_ stressor layer was also reported [23]. Through stressor capping of 2D materials [33, 34, 35], compressive or tensile strain from low to high levels can be achieved. Additionally, strain in 2D materials on pre‐patterned substrates, which were transferred mechanically on top of structured surfaces [36, 37, 38, 39, 40, 41, 42, 43], has achieved higher levels of strain up to ∼1%, but this approach has its own limitations, as detailed below.

Recently, we demonstrated that monolayer MoS_2_ can be strained through mechanical elongation on a sine‐patterned gate oxide using a polymer‐assisted transfer and pressing process, enabling the fabrication of strained MoS_2_ FETs that reach an electron mobility of 185 cm^2^/V·s with ∼1% tensile strain, showing an 8x improvement over unstrained counterparts [17]. Unlike sharp‐crested or rough patterns, grayscale topographies with smooth profiles promote better conformal attachment of 2D materials, mitigating wrinkling, rupture, and free‐standing suspended regions. Despite promising improvements, such approaches still face integration and scalability challenges due to defects, cracks, wrinkles, and contamination caused by the polymer‐assisted 2D material transfer from the growth substrate to the receiving target substrate, as shown in Figure S7. Furthermore, suspended 2D material flakes lead to unstable and non‐ideal semiconductor/dielectric interfaces.

To eliminate these issues, we propose a fundamentally new approach that introduces strain in 2D materials not after growth, but *during* growth directly on the target device having grayscale‐patterned surfaces instead of flat substrates. Here, we engineer device architectures with grayscale surface topography for in situ strain modulation in 2D materials grown on these pre‐patterned non‐planar nanotopographies. In this work, our new topography engineering approach is investigated for direct strain‐engineered growth of MoS_2_ on grayscale‐SiO_2_/silicon substrates for potential logic device scaling and on optically transparent grayscale‐SiO_2_/sapphire substrates for potential application innovations in the rapidly growing field of see‐through/transparent electronics and optics.

The main original contributions of our work are: (1) the strain‐engineered growth of 2D material films directly on the target substrates, which eliminates all transfer‐related issues of previous approaches known to introduce physical defects and contamination caused by polymer‐assisted transfer, as well as process uncertainties that result in different outcomes after each transfer process; (2) 3D/grayscale topography designs that allow deterministic and very localized strain engineering, such as bandgap gradients within the grown 2D monolayers, achieved in a well‐controlled, reproducible, and scalable way, as this approach relies on the CVD process rather than mechanical transfers; (3) the engineering of both strain levels and orientations through design, allowing them to be locally different if needed rather than being globally uniform; and (4) the potential combination with other methods to further enhance strain, as the source of strain in this work is based on device architecture through grayscale surface topography engineering.

## 2 Direct Strain‐Engineered Growth of 2D Materials

**FIGURE 1:**
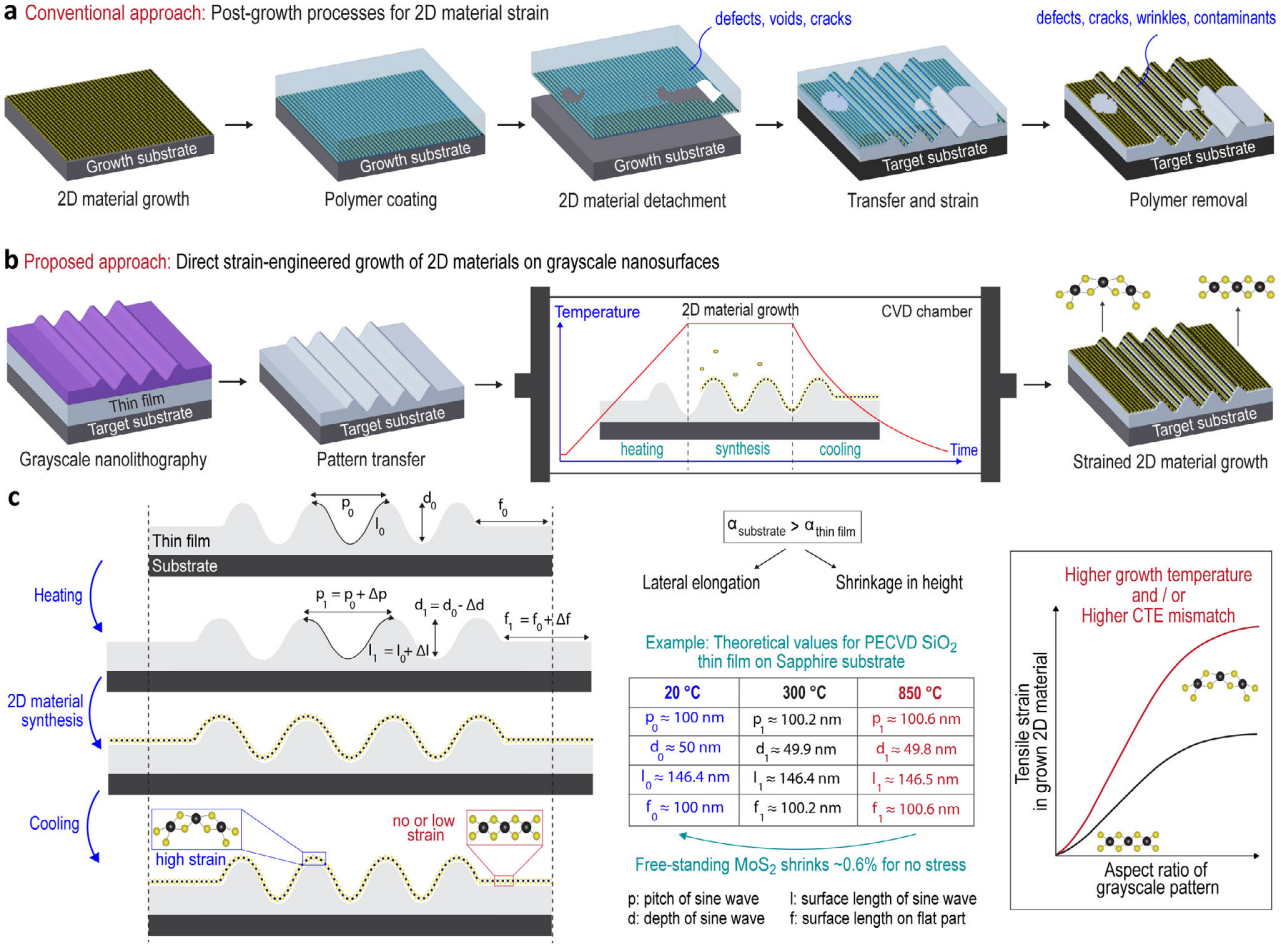
Conventional post‐growth strain vs. strain‐engineered growth on grayscale nanosurfaces. Illustration of (a) post‐growth processes for polymer‐assisted transfer‐based strain engineering of 2D materials, (b) direct strain engineering during growth, and (c) analytically calculated dimensions. The thermal expansion of the substrate influences the pitch, while tensile strain in the thin film induces shrinkage, reducing the depth. Under non‐slip conditions, the final length of the grown material matches the surface contour length of the underlying layer.

This fundamentally new direct strain‐engineered 2D material growth process relies on controlling surface contour length in grayscale‐patterned thin films during the heating, synthesis, and cooling steps. This approach is based on thermomechanical principles and requires three key conditions: (i) a substrate and at least one thin film must be present, composed of different materials, (ii) the coefficient of thermal expansion (CTE) of the substrate must differ from that of the thin film, with the substrate having a higher CTE for tensile strain, and (iii) the thin film must feature non‐planar structures. When the thermal expansion of the substrate is larger than that of the thin film in the stack, the heating phase, during which the stack is heated to 2D material growth temperatures, causes the CTE mismatch to produce two key effects in the thin film: lateral elongation due to substrate expansion and height shrinkage, which is proportional to the Poisson's ratio of the thin film and is caused by substrate‐induced in‐plane tensile stress. Critically, variations in surface contour lengths are constrained during both the heating and cooling steps within these grayscale segments of the thin film. After 2D material synthesis, during cooling, the 2D material that is firmly attached to the surface is forced to stretch (tensile strain) to match the length of the underlying surface morphology, under non‐slip conditions. Therefore, through the choice of materials in the stack and growth temperature, strain in the 2D material can be deterministically engineered (Figure 1).

This non‐slip assumption is supported by a previous study on strain engineering through thermal expansion mismatch [24], which discussed the importance of the adhesion between the 2D layer and the substrate being sufficiently strong and corrugated to prevent slipping, and which demonstrated both experimentally and analytically, that even atomic‐scale corrugation is sufficient to maintain strain in the grown material. Accordingly, the pre‐patterned thin films in this work, fabricated via lithography and plasma dry etching‐based pattern transfer, exhibit surface corrugation sufficient to prevent slippage between the 2D material and the underlying dielectric surface.

To quantify our hypothesis, Figure 1c shows analytical calculations for sine‐patterned plasma‐enhanced chemical vapor deposition (PECVD) SiO_2_ with a pitch of 100 nm and a depth of 50 nm on a c‐plane sapphire substrate. With increasing temperature, the pitch ($p_{1}$) increases by approximately 0.6% at 850 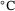 due to the higher in‐plane CTE of the substrate, while the depth ($d_{1}$) becomes approximately 0.4% shallower due to tensile stress‐induced shrinkage in the thin film. Because the out‐of‐plane thermal expansion of SiO_2_ is much smaller than the vertical compression, the net effect is a reduction in depth. Notably, while flat segments of the thin film surface experience contour length variation of about 0.6%, the grayscale segments show no significant contour length change. As a result, MoS_2_ grown at 850 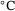 would shrink by approximately 0.6% during cooling under stress‐free conditions, but MoS_2_ grown on sine‐patterned areas does not shrink due to firm attachment, meaning its final length at room temperature is determined by the underlying sinusoidal profile length. This results in tensile strain in the MoS_2_ monolayer on pre‐patterned grayscale‐SiO_2_/sapphire, while the flat region remains strain‐free due to the close match between the thermal expansion coefficients of MoS_2_ and the c‐plane sapphire wafer [44, 45].

Grayscale thin‐film/substrate stacks are precisely engineered based on their thermal and mechanical properties. The level of strain depends on the aspect ratio of the grayscale nanotopographies, enabling deterministic strain control in the grown 2D material, which conforms to the surface morphology. According to this hypothesis, higher CTE mismatches and/or higher growth temperatures lead to more pronounced strain modulation in the grown 2D materials. Therefore, one of the main reasons for focusing on SiO_2_ thin films in this work is their remarkably low CTE, as a higher CTE mismatch is advantageous for increasing the induced strain level in addition to aspect ratio of grayscale patterns. See Supporting Information for details of the theory and Figures S1 and S2. Moreover, topographies with rounded, curved, or angled side profiles, including sinusoidal and triangular patterns, exploit these effects and induce strain in grown 2D materials similarly. Furthermore, the design of the topographies controls the orientation (e.g., uniaxial, multiaxial) of the strain. This approach enables different levels of strain‐induced bandgap modulation to be achieved within the same substrate. It also provides precise control over both the optical bandgap and the gradient of intermediate bandgap values, supporting applications that require engineered bandgaps for specific wavelength emission and absorption.

## 3 Scalable Grayscale Nanopatterning

For precise grayscale nanopatterning of SiO_2_ thin films, we used thermal scanning probe lithography (t‐SPL), which stands out with sub‐1 nm vertical and sub‐10 nm spatial resolution compared to other common techniques, such as electron beam lithography [46] and interference lithography [47]. t‐SPL utilizes a heated silicon nanotip to locally sublimate thermally sensitive resists like polyphthalaldehyde (PPA), achieving single‐digit nanometer resolution. The rapid and direct sublimation of PPA eliminates the need for wet development and minimizes thermal spreading effects from the tip through its endothermic decomposition, enabling high‐resolution patterning. Furthermore, t‐SPL's integrated write‐and‐read mechanism, featuring an embedded heater and thermal topography sensor, enables closed‐loop lithography, providing real‐time depth correction for sub‐nanometer vertical resolution in grayscale nanopatterning. Despite its strengths, key challenges include a practical patterning depth limit of typically less than 100 nm and an aspect ratio below 0.2 in grayscale nanopatterns written in PPA, as detailed in our previous publication [48], a low throughput from tip‐based scanning [49], and substrate constraints imposed by electrostatic actuation [50]. To address these challenges and achieve higher depths and aspect ratios, we exploit our previously developed etch amplification approach [48] and replication via nanoimprint lithography (NIL) for improved throughput and substrate versatility.

**FIGURE 2:**
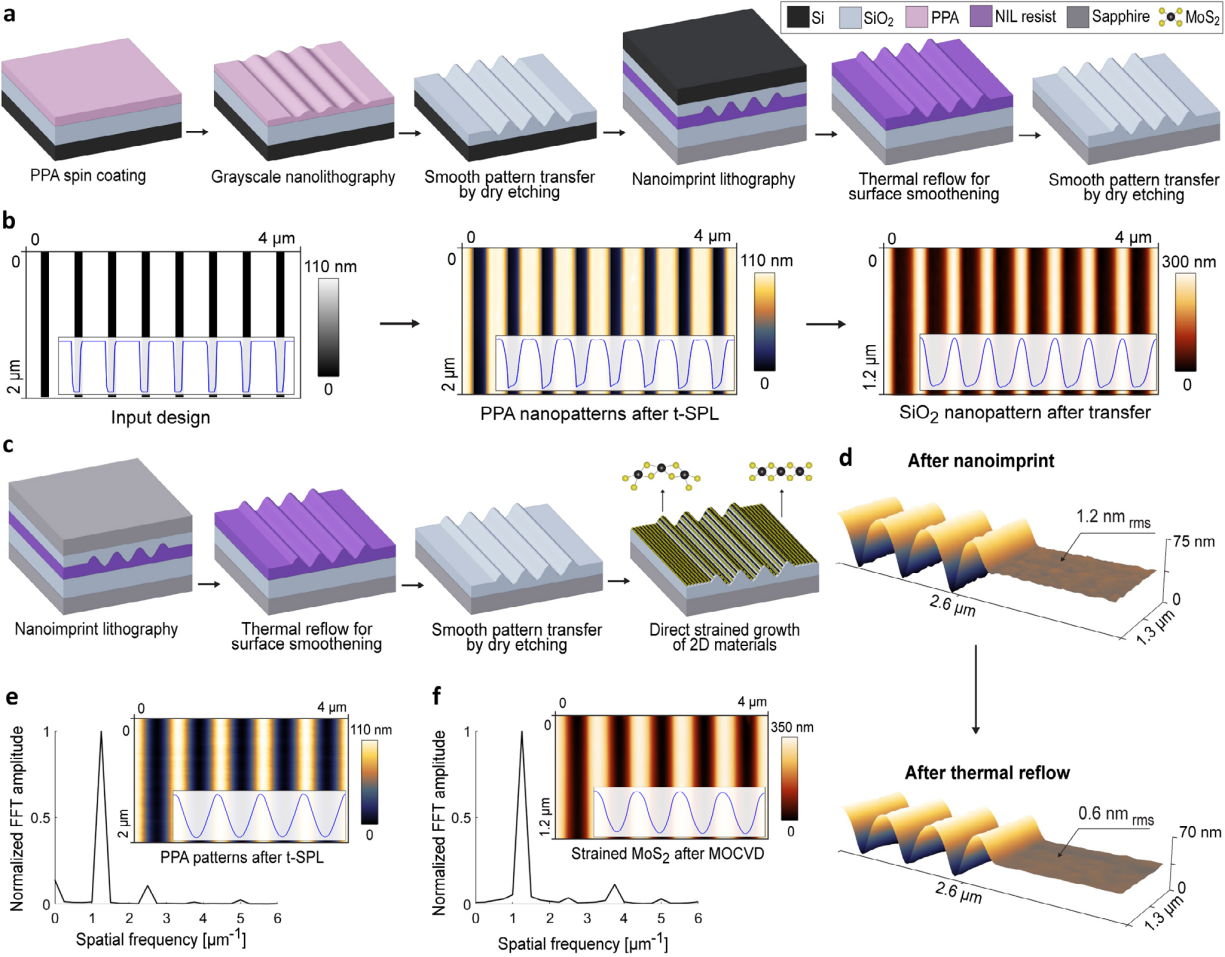
Scalable grayscale nanopatterning and direct strained growth. (a) Illustration of the fabrication process flow for grayscale nanopatterning, including stamp fabrication and the first replication step. (b) Strategy for fabricating sinusoidal nanopatterned stamps with a 500 nm pitch: a rectangular input design leads to trapezoidal PPA polymer patterns due to the t‐SPL tip geometry, which are then transformed into sinusoidal profiles through lateral etching during pattern transfer. (c) Illustration of the second nanoreplication step and 2D material growth for direct strain engineering. (d) Surface smoothing by thermal reflow to achieve sub‐1 ${\mathrm{nm}}_{\text{rms}}$ roughness. Fourier transforms of the measured topographies are shown in (e) after t‐SPL patterning on PPA for 800 nm pitch sinusoidal patterns, representing the first fabrication step, and (f) after 2D material growth on the final substrate, representing the last fabrication step.

Building on these considerations, our fabrication process consists of three main steps: (i) high‐resolution patterning using t‐SPL, (ii) depth amplification of the shallow sinusoidal polymer nanopatterns into thin‐film dielectrics such as SiO_2_ via fluorine‐based gentle plasma dry etching with cyclic cooling and etching to preserve surface smoothness, and (iii) scalable replication of these grayscale nanosurfaces through NIL on various substrates, in our case SiO_2_/Si and SiO_2_/sapphire (Figure 2). The combination of t‐SPL with plasma dry etching for nanopattern amplification is an efficient approach to achieve deeper and higher‐aspect‐ratio grayscale nanopatterns. However, shape distortion occurs during grayscale pattern transfer into hard materials, especially at high amplification depths, due to lateral etching. This effect is mitigable but unavoidable in grayscale transfer and is mainly caused by prolonged etch durations at low RF bias powers [48]. Although the vertical etch rate significantly exceeds the lateral rate, lateral broadening cannot be fully eliminated, resulting in shape deformation and reduced peak‐to‐peak depth for deeper patterns.

To address this issue, we reverse‐engineered the pattern design to compensate for etch‐induced distortion. For instance, to achieve a sinusoidal nanopattern with a 500 nm pitch, we started with a rectangular input design. When written in PPA using t‐SPL, the tip's conical geometry transforms these patterns into trapezoidal shapes, which are then transferred into SiO_2_ with depth amplification, ultimately yielding sinusoidal profiles (Figure 2b and Figure S3). In contrast, for larger‐pitch patterns (≥800 nm), we used sinusoidal designs directly, as lateral etching causes negligible distortion in such low‐aspect‐ratio structures. Fourier transform analysis of the measured topographies after t‐SPL on PPA—the first step in fabrication—and after MOCVD of the 2D material on the patterned SiO_2_/sapphire—the final step in fabrication—was performed to confirm the sinusoidal nature of the profiles (Figure 2c,d). The results demonstrate that, despite multiple etching stages, the sinusoidal features are reliably transferred thanks to the robustness of the optimized fabrication process.

As an industrialized technology, particularly in optical metasurfaces, NIL offers an energy‐efficient, cost‐effective, and high‐throughput alternative to EUV lithography [51, 52, 53], aligning with the goal of sustainable and scalable manufacturing. Here, we use NIL for single‐digit nanometer precision grayscale nanopatterning, going beyond binary nanopatterning, and demonstrating its application in nanoelectronics. These grayscale SiO_2_/Si patterns are used as stamps to replicate nanostructures onto thermoplastic NIL resists across various substrates. Additionally, although optimized dry etching‐based grayscale pattern transfer recipes are refined to prevent additional surface roughness from the plasma process itself, roughness increases proportionally with depth amplification. To mitigate this, thermal reflow of the imprinted NIL polymer is performed by heating it near its glass transition temperature, effectively smoothing the surface. However, unlike the common reflow experiments reported in the literature, which are typically performed to round staircase features following grayscale electron beam lithography [52], we preserve the original topology and instead focus on smoothing the surface roughness introduced during the etching‐based depth amplification process. We demonstrate that a 3‐h heat treatment at 110 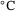, just below the NIL resist's glass transition temperature of 115 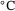, reduces surface roughness from >1 ${\mathrm{nm}}_{\text{rms}}$ to about 0.5 ${\mathrm{nm}}_{\text{rms}}$ (Figure 2e). In contrast, reflow above the glass transition temperature leads to significant and undesirable depth loss. Detailed information on the thermal reflow process is provided in the Figure S5.

Following heat treatment, the replicated polymer patterns are transferred into SiO_2_ via a second dry etching process, using the same gentle plasma recipe applied during the initial PPA‐to‐SiO_2_ transfer. The resulting grayscale‐patterned substrates are also used as stamps for a second NIL cycle, during which thermal reflow and etching are repeated to correct shape deformation and rounding effects, enabling the formation of a sinusoidal profile in the final substrate. These plasma recipes are broadly applicable and compatible with various polymer‐dielectric stacks, independent of the lithography technique employed. In the final patterned substrates used for the strained growth of 2D materials, we achieve a surface roughness of <1 ${\mathrm{nm}}_{\text{rms}}$ for sinusoidal nanopatterns with depths of up to 400 nm. The key strategy involves the following sequence: first, amplify the pattern depth by >3–4×; then perform NIL followed by thermal reflow and pattern transfer with a slight amplification of approximately 1.2×; finally, repeat the NIL, thermal reflow, and transfer steps with a similar ∼1.2× depth amplification. This fabrication process is repeated on different substrates such as silicon and sapphire and on various thin‐film oxides (i.e., thermally grown or CVD‐deposited films). It is known that roughness leads to nano‐ to micrometer scale cracks that cause failure of the final devices [17, 48], such as transistors, and can also induce highly localized and non‐homogeneous electric field distributions. Since surface smoothness is very important, it is systematically studied in this work.

## 4 Results and Discussion

To demonstrate that strain can be directly introduced into 2D materials during their growth on engineered grayscale nanotopographies, we perform metal–organic chemical vapor deposition (MOCVD) of MoS_2_ at 850 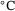 on sinusoidally nanopatterned oxide thin film surfaces, a scalable approach that ensures large‐area uniformity in both thickness and composition. The resulting strain‐induced bandgap modulation is experimentally confirmed through photoluminescence (PL) spectroscopy mapping across various MoS_2_/grayscale‐thin‐film/substrate stack configurations, providing clear experimental evidence supporting our hypothesis. To characterize the effect of strain, we use PL spectroscopy mapping, as strain in 2D semiconductors leads to shifts in the PL peak position. Compared to Raman spectroscopy, PL mapping offers higher sensitivity to unit strain, making it a more reliable and interpretable method for strain analysis in 2D materials.

**FIGURE 3:**
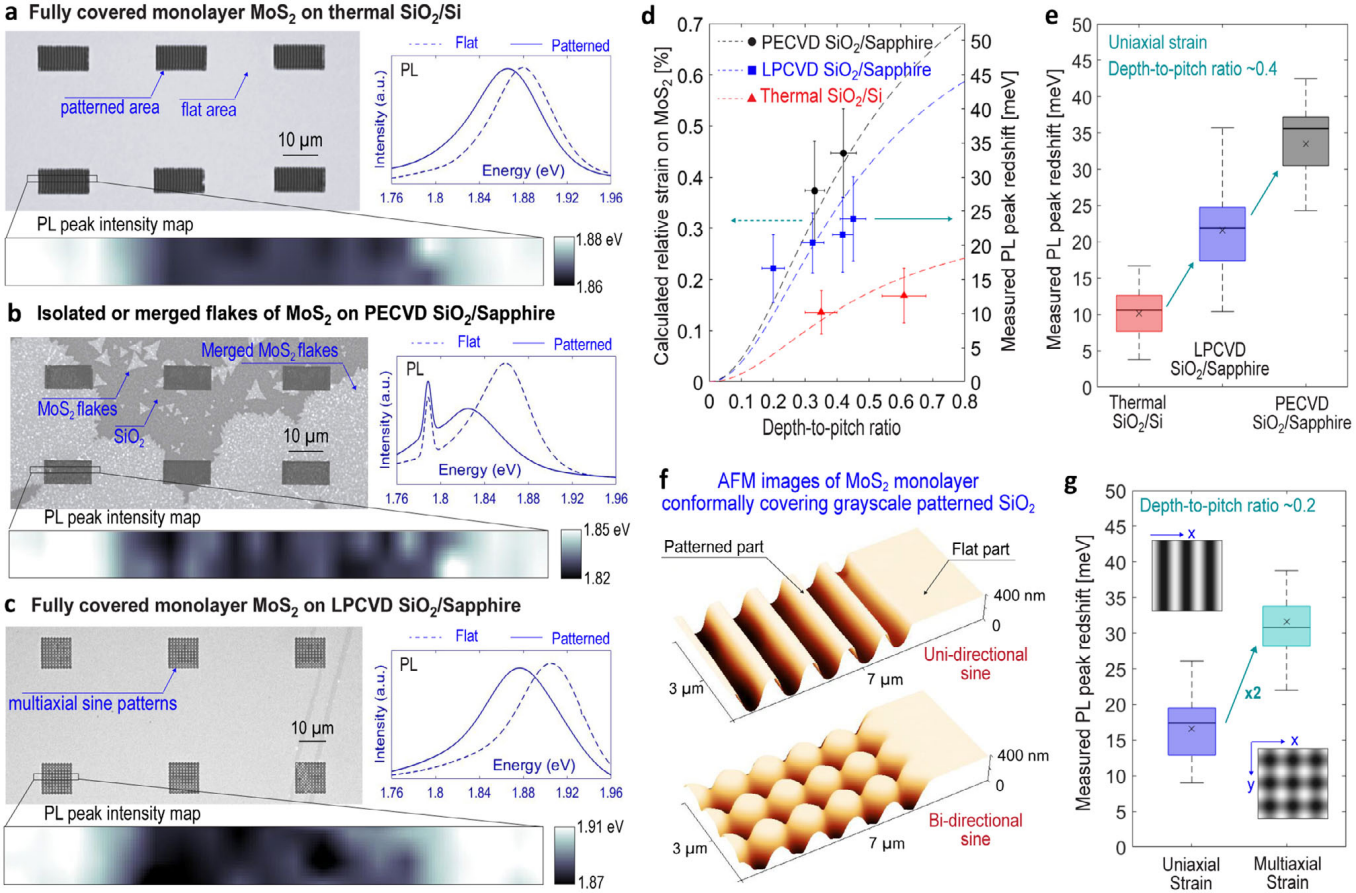
Strain characterization. Photoluminescence (PL) spectra and peak intensity mapping of MoS_2_ grown on flat and grayscale nanopatterns, fabricated on (a) thermal SiO_2_ on silicon, (b) PECVD SiO_2_ on sapphire, showing isolated triangular or merged flakes, and (c) LPCVD‐annealed TEOS‐based SiO_2_ on sapphire. (d) Calculated strain values (dashed line) and measured PL energy peak shifts as a function of depth‐to‐pitch ratio. The error bars represent the standard deviation, denoted as ±σ, based on PL measurements at 13 to 72 different positions. For the peak shift calculation, the flat unstrained peak position is averaged over 20 different points near the patterned area. (e) Box‐and‐whisker plot corresponding to a depth‐to‐pitch ratio of approximately 0.4. (f) AFM image showing conformal growth of MoS_2_ on sinusoidal patterns modulated in one or two directions. (g) Comparison of uniaxial and multiaxial strain at a depth‐to‐pitch ratio of ∼0.2.

We begin our experiments using grayscale‐patterned thermally grown SiO_2_ on silicon and plasma‐enhanced chemical vapor deposition (PECVD) SiO_2_ deposited at 320 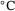 on c‐plane sapphire substrates to experimentally demonstrate how different substrate stacks induce different strain levels, following the fabrication process detailed above. On grayscale‐patterned thermal SiO_2_, we obtain continuous MoS_2_ monolayer films, that are subsequently characterized by PL and Raman spectroscopy mapping (Figure S9) that confirm the expected strain‐induced bandgap modulation (Figure 3a). On the other hand, MoS_2_ grown on PECVD SiO_2_ surfaces typically forms isolated triangular flakes or merged few‐layer structures rather than uniform monolayer films (Figure 3b). The PL signal in these cases is weaker and broader, with a full width at half maximum (FWHM) of 86 meV compared to 75 meV on thermal oxide. As shown in Figure S8, AFM images reveal that even in the best‐performing PECVD chips, hole‐like defects are present, and in most cases, the sinusoidal pattern is severely deformed. We believe that this is due to precursor diffusion into the lower‐density PECVD oxide [54], which hinders the growth of a monolayer of the 2D material on the surface. To improve surface quality, we applied atomic layer deposition (ALD) of a thin SiO_2_ or Al_2_O_3_ capping layer (10–20 nm); however, surface deformation still occurred after MOCVD.

To overcome this issue, we densified the SiO_2_ thin film by modifying the deposition method and introducing high‐temperature heat treatments. Specifically, we fabricated grayscale nanopatterns on low‐pressure chemical vapor deposition (LPCVD) tetraethoxysilane (TEOS)‐based SiO_2_, deposited at 725 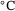, followed by thermal annealing at 1050 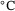 in an O_2_ atmosphere for 2 h. A second annealing step consisting of a 3‐h treatment at 1100 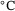 in an Ar atmosphere was performed after grayscale patterning of these oxide films that had previously been heat‐treated. As shown in Figure 3c and Figure S10, this approach yields continuous MoS_2_ monolayers on the patterned surface, comparable to those grown on thermal oxide. Consistent with these observations, the PL spectra exhibit a stronger intensity peak and a narrower FWHM of 74 meV, matching with the thermal SiO_2_ reference case, thus confirming high‐quality strain‐engineered growth. The slightly broader peaks can be related to roughness, as surface roughness is typically 0.5–1 ${\mathrm{nm}}_{\text{rms}}$ on the grayscale‐patterned regions, while it is generally 0.5–0.7 ${\mathrm{nm}}_{\text{rms}}$ on flat regions. The FWHM of the PL peak on the flat region is 68 meV, which can be related to a slightly lower defect density compared to the grayscale regions [55]. However, these values are still lower than previously observed FWHM values of exfoliated or CVD‐grown MoS_2_ monolayers [29, 36], demonstrating the potential of our approach for low‐defect, transfer‐free integration of these materials. Finally, by comparing PL peak positions between flat and structured areas in the different material stacks, we demonstrate that the observed shifts in MoS_2_, corresponding to 75 meV/% of uniaxial strain, correlate well with our analytical strain calculations, as detailed in the Supporting Information.

The induced tensile strain on the patterned regions primarily results from the CTE mismatch between the thin film and the substrate, SiO_2_/Si and SiO_2_/sapphire stacks in our case. As expected, larger CTE mismatches produce higher strain levels. Densification of the thin films is also critical for achieving high‐quality material growth, especially when targeting fully covered, large‐area MoS_2_ monolayers. The strain magnitude observed in densified LPCVD‐annealed TEOS‐based SiO_2_ is slightly lower than that of PECVD SiO_2_, which is attributed to its lower Poisson's ratio. This lower Poisson's ratio affects the depth of the pre‐patterned topographies at high temperature and influences the induced strain in the grown material after cooling. On the other hand, importantly, the depth‐to‐pitch ratio of sinusoidal nanopatterns in the SiO_2_ layer enables deterministic control of the strain level. By precisely adjusting the aspect ratio of these grayscale topographies, we demonstrate strain levels ranging from 0 to approximately 0.5% average tensile strain in the grown material (Figure 3), and show that it is possible to achieve strain up to approximately 1% tensile strain on sapphire substrates at higher depth‐to‐pitch ratios, as also predicted analytically (Figure S2).

Furthermore, beyond controlling strain magnitude, the orientation of strain can also be controlled through the precise grayscale nanopatterning capability of t‐SPL. In this study, in addition to a unidirectional sinusoidal design, we pattern sinusoidal wave nanopatterns modulated in two directions: f(x,y) = *A*[cos(gx)+cos(gy)], where A and g are the amplitude and spatial frequency, respectively, to achieve multiaxial strain in grown 2D materials. Consistent with prior research [56], we show that strain‐induced bandgap modulation is 2× as efficient for multiaxial strain compared to uniaxial strain (Figure 3h). This multiaxial strain results in a ∼32 meV PL peak shift for an aspect ratio of 0.2, whereas it is only ∼17 meV for uniaxial strain. These values demonstrate that the extracted bandgap modulation corresponds to ∼150 meV/% under multiaxial strain, as obtained from first‐principles calculations of the variation in valley energy separation as a function of the applied strain, in agreement with previous experimental results [17].

**FIGURE 4:**
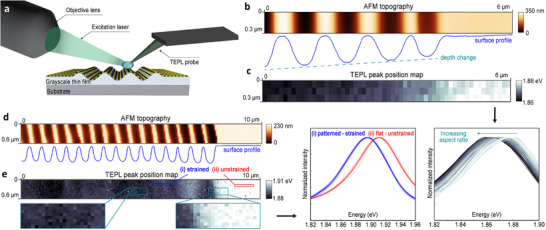
Nanoscale TEPL characterization of strain‐induced bandgap modulation. (a) Schematic illustration of the side‐illumination tip‐enhanced photoluminescence (TEPL) setup used for hyperspectral nanoscale photoluminescence imaging via plasmonic near‐field enhancement. (b) AFM topography and (c) TEPL peak position map of MoS_2_ grown on a SiO_2_/Si substrate with sinusoidal patterns of gradually increasing depth and aspect ratio, demonstrating controlled strain‐induced bandgap modulation. The inset shows the full cross‐sectional topography profile. TEPL measurements were performed with a 100 nm step size across the 6 μm sample length, and the plot displays 60 spectra measured along the third row of the TEPL map. (d) AFM topography and (e) TEPL peak position map of MoS_2_ spanning both the flat and grayscale nanopatterned regions of the SiO_2_/sapphire substrate. The TEPL map was acquired with a 50 nm step size. The inset shows the entire cross‐sectional topography profile. Magnified views highlight the strained region extending across multiple 500 nm‐pitch sine waves and the transition zone from flat to patterned areas. Representative TEPL spectra from 20 measurement points along a 1 μm line are shown for both flat (red) and patterned (blue) regions, with solid lines indicating averaged curves. See the Supporting Information for detailed comparisons of the materials grown on thermal and LPCVD SiO_2_ surfaces.

In addition to far‐field PL mapping, we employed nanoscale tip‐enhanced photoluminescence (TEPL) imaging, where a silver‐coated AFM tip is combined with PL spectroscopy to induce localized plasmonic enhancement (Figure 4a). This method enables direct strain characterization in monolayer TMDs with spatial resolution better than 20 nm [57, 58]. In this nanoscale characterization, as theoretically expected, the induced strain is spatially distributed across the entire patterned area (Figure 4d, e), and its level is controlled by the grayscale surface topography. The 2D material conformally follows the surface contours at high temperatures during the growth, and the changes in surface length during cooling are critical to strain formation. Thus, strain‐induced bandgap engineering in 2D semiconductors in this work occurs across the entire patterned surface, not just at morphological variations such as crests and valleys. Importantly, the strain distribution is highly uniform across each sine wave, with no measurable difference in strain between crests and valleys. Specifically, as the CTE of the substrate exceeds that of the thin film, we observe fully tensile strain across all patterned areas.

Additionally, by controlling the depth‐to‐pitch ratio within the same patterned area through precise depth modulation during t‐SPL patterning, as shown in Figure 4b, it is possible to achieve precise bandgap control with a resolution down to 1 meV. To study this effect systematically, we intentionally examined strain on a sinusoidal nanopattern with a gradually increasing aspect ratio by increasing the depth while maintaining the same periodicity in the design. As expected, we observed a corresponding gradual reduction in the bandgap due to tensile strain (Figure 4c), which enables deterministic control of strain‐dependent material properties within the same device. Such precise grayscale control provides deterministic bandgap engineering through strain, potentially enabling a new generation of devices with customized optical and electronic properties not available in as‐grown materials. These results are fundamentally enabled by the deep, smooth, and high‐resolution sinusoidal nanopatterning developed in this study, which highlights the critical importance of grayscale nanopatterning.

Most research focuses on achieving higher‐quality 2D material growth by mitigating intrinsic disorders such as point defects, vacancies, and atomic impurities to enhance uniformity over large areas. However, material transfer from the growth substrate to the target substrate (detachment, alignment, and transfer) undermines these improvements in 2D material quality, as it typically introduces extrinsic defects, including contamination, and physical damage such as cracks, wrinkles, voids, and ruptures, all of which degrade device performance. These issues lead to non‐ideal semiconductor/dielectric interfaces, primarily due to interface traps and polymer contamination that significantly affect device behavior and performance. Importantly, the transfer‐free approach introduced in this work ensures reproducibility with full conformal attachment of the grown MoS_2_ onto the dielectric, as confirmed by AFM topography maps. Clean 2D semiconductor/dielectric interfaces are essential for CMOS applications, as well as for other electronic and optical applications, to enhance device reliability (Tables S1 and S2).

Direct growth of 2D materials on non‐planar surfaces has been performed previously, but for sidewall transistors with the purpose of increasing transistor density per unit area rather than for strain engineering [59]. We employ grayscale topographies to enable the 3D vertical stacking of atomically thin materials and their strain‐induced modification. It should be noted that, as previously mentioned, grayscale topographies are not limited to sinusoidal profiles. 2D material growth on any rounded or inclined surfaces, such as the fin shapes commonly used in nanoelectronics for FinFETs, can significantly benefit from this approach. Consequently, the grayscale‐thin‐film/substrate stack can be engineered to predetermine both the magnitude and orientation of strain, depending on application requirements. It has been experimentally and analytically demonstrated that tensile‐strained MoS_2_ transistors exhibit remarkable enhancements in electron mobility, especially within the first 0.5% of applied strain, where the most pronounced improvement compared to unstrained devices is typically observed [17, 60]. Based on these results, mobilities of ≥100 cm^2^/V·s appear achievable on grayscale‐patterned SiO_2_/Si or SiO_2_/sapphire stacks [17].

Moreover, the approach is adaptable to a wide range of material combinations. For example, using high‐CTE polymers with a grayscale‐patterned oxide (e.g., MoS_2_/grayscale‐SiO_2_/polyimide stacks) enables tensile‐strained MoS_2_ in patterned regions, even at low processing temperatures, while maintaining compressive strain in flat areas. This versatility, combined with recent advancements in low‐temperature growth of 2D materials [12, 61], makes this approach potentially attractive for back‐end‐of‐line (BEOL) integration in CMOS circuits (<400 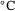), as well as for integration into flexible electronics and polymer‐based optical applications at compatible temperatures, which cannot be achieved with conventional CVD approaches due to the high thermal budget required. See the Supporting Information for a detailed explanation of the effect of temperature on the induced strain levels in the grown material (Figure S1).

Beyond scaling, introducing optical transparency at the device level could enable novel applications aligned with the *More‐than‐Moore* vision. One such opportunity lies in the rapidly growing field of see‐through/transparent electronics, which could significantly benefit from the integration of see‐through transistors, unachievable with conventional silicon‐based technologies. We study transmittance on strain‐engineered MoS_2_ on sapphire substrate, as shown in (Figure S11). Such transparent yet high‐performance semiconductors are promising candidates for next‐generation applications in displays, wearable electronics, optical sensors, and human‐machine interfaces.

## 5 Conclusion

In conclusion, through the direct strain‐engineered growth approach proposed in this work, we deterministically control both the strain levels (experimentally ranging from 0 to 0.5% tensile strain, with higher values theoretically possible) and orientations (uniaxial and multiaxial) in MoS_2_ during its growth on pre‐determined sine‐shaped SiO_2_ surfaces on either Si or sapphire substrates. Unlike post‐growth 2D material strain, we utilize pre‐growth nanopatterning to engineer the surface topographies of the substrate stack and introduce strain directly during the growth process. This strain technique is broadly applicable to any 2D semiconductor expected to benefit from strain and is general enough to be applied to any 2D material/grayscale‐thin‐film/substrate stack, including flexible and transparent platforms. Furthermore, as topography engineering serves as an efficient primary strain source, it can be combined with additional methods such as dielectric stressor capping or doping to further enhance strain.

The presented strain approach proposes potential innovation in device architecture through grayscale surface topography engineering that introduces strain into the 2D semiconducting channels and improves their performance for future semiconductor scaling and innovation. This transfer‐free strain‐engineering method eliminates the need for polymer‐assisted material transfer from the growth substrate, thus avoiding defects, cracks, wrinkles, and contamination. This results in a reliably reproducible, scalable, and industry‐compatible process that ensures full conformal attachment of the 2D semiconductor to the underlying dielectric. It enables stable, air‐trap‐free and contamination‐free semiconductor/dielectric interfaces, essential for advanced nanoelectronics and optical applications. The demonstrated strain‐engineered 2D semiconductor on silicon substrates represents a contribution to CMOS logic device scaling with potential for the integration of 2D semiconductors. In parallel, the relatively high strain achieved on sapphire substrates holds promise for further transistor scaling and fast sensor applications, as well as for enabling large‐area transparent electronics and optoelectronic devices that require precise bandgap engineering for targeted light emission and absorption, achieved through material innovation at the device level. Consequently, the proposed technique extends beyond traditional material engineering, presenting a versatile solution for a broad spectrum of electronic and photonic applications.

## 6 Methods

### 6.1 Grayscale Nanopatterning

A commercial t‐SPL system (Nanofrazor Explore) and thermal cantilevers of type NanoFrazor Monopede (Heidelberg Instruments Nano AG) were used to pattern grayscale nanostructures on PPA. For resist coating, a 7.5 wt.% solution of polyphthalaldehyde (PPA, Allresist) in anisole (Sigma–Aldrich Chemie GmbH) was spin‐coated at 4000 rpm on 500 nm thick thermal ${\text{SiO}}_{2}$/Si substrates and soft‐baked at 110 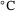 for 2 min. For grayscale design, the analytical designs of sinusoidal patterns were converted into a grayscale bitmap consisting of a 20×20 ${\text{nm}}^{2}$ pixel grid with a normalized depth of 256 levels. The minimum depth (white pixel) and maximum depth (black pixel) of the grayscale bitmap images were assigned in NanoFrazor: “binary writing” mode for rectangular designs and 10 to 110 nm depths for sinusoidal designs. For t‐SPL, the writing heater temperature was set to 1050 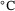, the step size to 20 nm, the scan speed to 25 μs per pixel, and the force pulse to 5 μs. The patterned depths were simultaneously corrected by integrated in situ AFM metrology, with NanoFrazor's PID feedback used for rectangular writing and Kalman feedback for sinusoidal writing to adjust the actuation forces for high‐resolution depth control.

For plasma‐based pattern transfer, PPA nanopatterns were transferred into the ${\text{SiO}}_{2}$ layer on a Si substrate using an ICP‐based RIE system (SPTS Advanced Plasma System) with ${\text{CHF}}_{3}$/${\text{SF}}_{6}$ plasma at a flow rate of 50/10 sccm, 950 W RF ICP power, 18 W RF bias power, 5 mTorr pressure, and an initial substrate temperature of 10 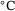. Every 100 s, the plasma was turned off for 300 s to lower the substrate temperature. Meanwhile, Ar was introduced into the plasma chamber to accelerate substrate cooling. We refer to this as a cycled cooling‐etching process. For cleaning the grayscale surfaces, a piranha solution (3:1 mixture of $H_{2}{\text{SO}}_{4}$ (96%) and $H_{2}O_{2}$ (30%)) was used for 10 min, followed by sequential cleaning in acetone (5 min), IPA (5 min), and $O_{2}$ plasma (10 min). Since the nanostructured substrates are used as NIL stamps, 1H,1H,2H,2H‐perfluorooctyltrichlorosilane was coated in vapor phase for 10 min to silanize the SiO_2_ stamp, facilitating easy release after imprinting. We observed that the stamp can withstand over 10 uses with acetone and IPA cleaning. After 10 replications, we recommend cleaning the stamp surface with $O_{2}$ plasma and re‐silanizing it for continued use.

For nanoreplication through NIL, the thermoplastic resist mr‐I 8030R (micro resist technology GmbH) was spin‐coated on target substrates of thermal ${\text{SiO}}_{2}$/Si, PECVD ${\text{SiO}}_{2}$/sapphire, or LPCVD+annealed ${\text{SiO}}_{2}$/sapphire at 1600 rpm. The grown or deposited ${\text{SiO}}_{2}$ thin films are 1 μm thick. A NILT CNI v3.0 tool from NIL Technology (NILT) was used for the replication of grayscale nanostructures. The NIL process was performed with a temperature ramp‐up of 9 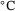/min to 200 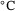 under 2 bar pressure, followed by 60 min at 200 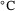 under 6.8 bar pressure. The sample was then cooled down to 85 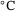 without pressure and manually detached. The imprinted NIL polymer is thermally reflowed in an oven at 110 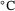 for 3 h. The polymer nanopatterns were transferred into ${\text{SiO}}_{2}$ using ${\text{CHF}}_{3}$/${\text{SF}}_{6}$ plasma at a flow rate of 50/10 sccm, 950 W RF ICP power, 70 W RF bias power, 5 mTorr pressure, and an initial substrate temperature of 10 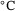. The plasma was turned off for 300 s after every 100 s. For some chips, these patterned substrates were used as intermediate stamps and replicated again. In the case of intermediate stamps, since the bottom flat part of the imprinted polymer reaches the sapphire substrate, 10 nm of SiO_2_ was deposited by ALD to fully cover the surface with the same material, SiO_2_. This approach aimed to obtain a thicker dielectric height on the flat region, depending on the design. The same NIL and dry etching processes were repeated. Again, for cleaning the grayscale surfaces, a piranha solution (3:1 mixture of $H_{2}{\text{SO}}_{4}$ (96%) and $H_{2}O_{2}$ (30%)) was used for 10 min, followed by sequential cleaning in acetone (5 min), IPA (5 min), and $O_{2}$ plasma (10 min).

### 6.2 2D Material Growth

Large‐area MoS_2_ films were synthesized by MOCVD. An aqueous solution of sodium molybdate Na_2_MoO_4_ was spin‐coated onto the pre‐patterned substrate, serving as the molybdenum (Mo) source. The coated samples were then loaded into the CVD chamber, and diethyl sulfide (C_2_H_5_)_2_S was introduced into the quartz tube as the sulfur (S) precursor with argon and hydrogen as the carrier gases. The chamber temperature was ramped to 850 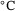 over 80 min, and the growth reaction proceeded at this temperature for approximately 30 min.

### 6.3 Metrology and Strain Characterization

AFM topography characterization was performed using a Bruker FastScan AFM (ScanAsyst mode). ScanAsyst auto control was used as the feedback system, and the step size in topography imaging was set to 20 nm. For data visualization and surface profile characterization, the scanning probe analysis software Gwyddion (version 2.59) was used. Data plotting and Fourier transforms of patterns were performed in MATLAB (version R2020b). RMS surface roughness on flat areas was quantified as the mean value over the region of interest. RMS surface roughness on sinusoidal areas was calculated by subtracting the mean sinusoidal profile from the measured topographies. For the PL spectra, a Renishaw inVia Reflex Raman Confocal Microscope was utilized. The data were collected by averaging two accumulations of 1 s laser exposure with an excitation wavelength of 532 nm. A grating of 300 gr/mm was used. To avoid damage to the studied samples, the laser power was kept at ≤35μW. For micro‐PL maps, step size of 1 μm is used and values are interpolated in the plots.

To prepare TEPL probes, Si AFM cantilevers (Nanosensors, Switzerland) were first oxidized in a furnace (Carbolite Gero, UK) at 1000 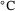 for 23 h to increase the refractive index of the surface, followed by UV‐ozone (Ossila, UK) cleaning for 1 h. The cleaned probes were placed in a thermal evaporation chamber of a N_2_ glovebox (MBraun, Germany). The AFM cantilevers were coated with a 150 nm thick layer of Ag (Advent Research Materials, UK) at a rate of 0.5 nm/s under 10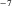 mbar pressure. TEPL measurements were performed under ambient conditions using a side‐illumination system consisting of a Raman spectrometer (HORIBA Scientific, France) and an atomic force microscope (AIST‐NT, USA). 532 nm excitation laser was incident on the sample at an angle of 60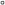 with respect to the surface and focused on the sample using a 100×, 0.7 NA objective lens (Mitutoyo, Japan). TEPL line mapping was performed using a step size of 50 or 100 nm, spectrum acquisition time of between 0.1 to 1 s and a laser power of 118 μW at the sample. TEPL spectra were collected using a spectrometer grating of 300 lines/mm and a CCD detector. For TEPL maps, no interpolation is applied to the extracted data.

## Author Contributions

B.E., G.B., A.K. and J.B. conceived and developed the idea of direct strained growth on grayscale surfaces, as well as the experiments for fabricating and characterizing strain‐engineered materials. B.E., with the supervision of G.B. and J.B., performed sample preparation, t‐SPL, NIL, dry etching processes, theoretical calculations, optical measurements, metrology characterizations, and data analysis. A.B. and A.K. conceived and developed the MoS_2_ growth process. A.B. performed the MOCVD. B.E., A.D. and N.K. performed TEPL and data analysis. B.E., G.B., and J.B. wrote the manuscript with input from all authors.

## Conflicts of Interest

The authors declare no conflicts of interest.

## Supporting information


**Supporting File**: advs74212‐sup‐0001‐SuppMat.pdf.

## Data Availability

The data and code of this study are available on Zenodo at https://doi.org/10.5281/zenodo.18432579.
