# Supplementary material for: Engineered Strain in 2D Materials by Direct Growth on Deterministically Patterned Grayscale Topographies
Source: Adv Sci (Weinh). 2026 Feb 10;13(20):e22850. doi: 10.1002/advs.202522850 (PMC13067839; doi:10.1002/advs.202522850)
Supplement: Supplementary file 1 — Supporting File: advs74212‐sup‐0001‐SuppMat.pdf. [file ADVS-13-e22850-s001.pdf]

# – Supplementary Information –

Engineered Strain in 2D Materials by Direct Growth on Deterministically Patterned Grayscale Topographies

Berke Erbas, Arindam Bala, Hernan Furci, Anushree Dutta, Naresh Kumar, Renato Zenobi, Giovanni Boero, Andras Kis, Juergen Brugger

S1 Analytical calculations for strained 2D material growth

The details of analytical calculations for strained 2D material growth, which take into account the engineering of substrate stacks with grayscale topographies to induce strain in the 2D material grown on the surface contours, are explained below.

Sine profile at room temperature:

$$z_0(x) = \frac{d_0}{2}\cos\left(2\pi\frac{x}{p_0}\right) \tag{S1}$$

The initial length of sine profile at room temperature:

$$l_0 = \int_0^{p_0} \sqrt{1 + \left(z_0'(x)\right)^2}\,dx \tag{S2}$$

$$z_0'(x) = \frac{d}{d_x} z_0(x) \tag{S3}$$

The pitch of sine wave at growth temperature:

$$p_1 = p_0\left(1 + \alpha_{substrate}\Delta T\right) \tag{S4}$$

Strain in horizontal direction due to thermal expansion mismatch between substrate and thin film layer:

$$\varepsilon_x = \frac{p_1 - p_0}{p_0} = \alpha_{substrate}\Delta T \tag{S5}$$

Height change in thin film due to stress:

$$\varepsilon_z = \frac{d_1 - d_0}{d_0} = \left(\frac{-2\nu}{1-\nu}\right)\varepsilon_x \tag{S6}$$

The total depth at the growth temperature, accounting for height shrinkage due to stress and thermal expansion:

$$d_1 = d_0\left(1 - \frac{2\nu}{1-\nu}\varepsilon_x\right) + d_0\alpha_{thin-film}\Delta T \tag{S7}$$

Sine profile at growth temperature:

$$z_1(x) = \frac{d_1}{2}\cos\left(2\pi\frac{x}{p_1}\right) \tag{S8}$$

The length of sine profile at growth temperature:

$$l_1 = \int_0^{p_1}\sqrt{1 + \left(z_1'(x)\right)^2}dx \tag{S9}$$

$$z_1'(x) = \frac{d}{d_x}z_1(x) \tag{S10}$$

The length of grown 2D material:

$$l_{2D\rightarrow T_{growth}} = l_1 \tag{S11}$$

For non-slip condition assumption:

$$l_{2D\rightarrow final} = l_0 \tag{S12}$$

Free-standing 2D material, grown at high temperature and cooled to room temperature, should result in zero strain for following length:

$$l_{2D\rightarrow no\ stress} = l_{2D\rightarrow T_{growth}}\left(1 + \alpha_{2D}\Delta T\right) \tag{S13}$$

Strain in 2D material after cooling down:

$$\varepsilon_{2D} = \frac{l_{2D\rightarrow final} - l_{2D\rightarrow no\ stress}}{l_{2D\rightarrow no\ stress}} \tag{S14}$$

In this work, averaged linear thermal expansion coefficients over the temperature range of 20 to 850 °C are considered: $\alpha_{\mathrm{MoS_2}} \approx \alpha_{\mathrm{sapphire}} = 7.5\times10^{-6}$/°C > $\alpha_{\mathrm{Si}} = 3.3\times10^{-6}$/°C > $\alpha_{\mathrm{SiO_2}} = 0.55\times10^{-6}$/°C. In addition, Poisson's ratios of $\nu_{\mathrm{PECVD\ SiO_2}} = 0.25 > \nu_{\mathrm{TEOS\ SiO_2}} = \nu_{\mathrm{Thermal\ SiO_2}} = 0.17$ are used. Although the out-of-plane thermal expansion of the SiO_2 thin film is minimal compared to stress-induced height shrinkage, it is still included in the analytical calculations to generalize the formula for potential use with high-CTE thin films.

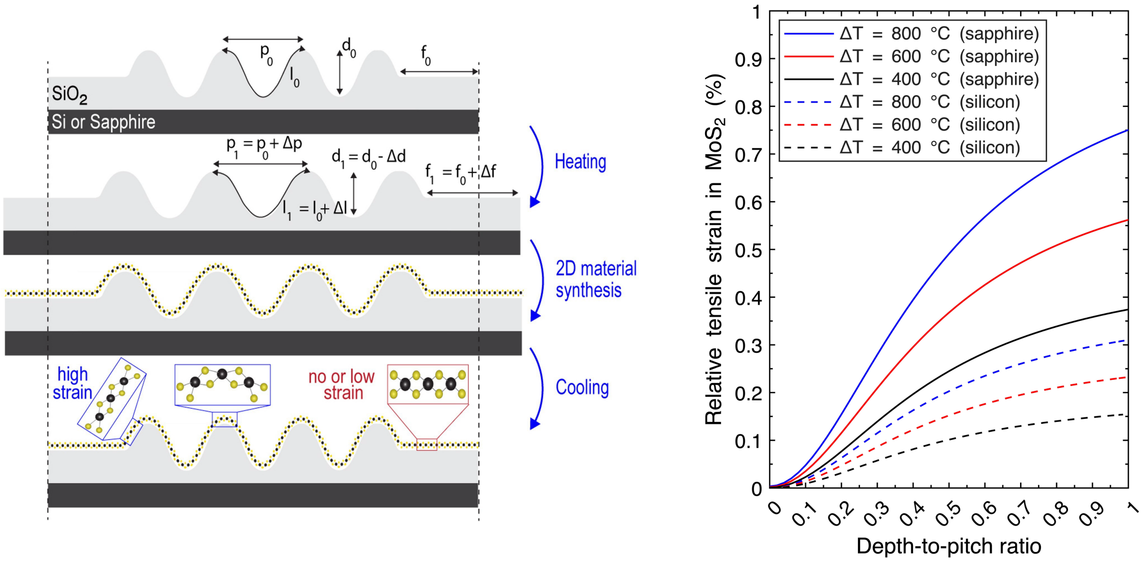

Figure S1: The effect of growth temperature on the induced strain in the grown 2D material on patterned PECVD SiO_2 on either Si or sapphire.

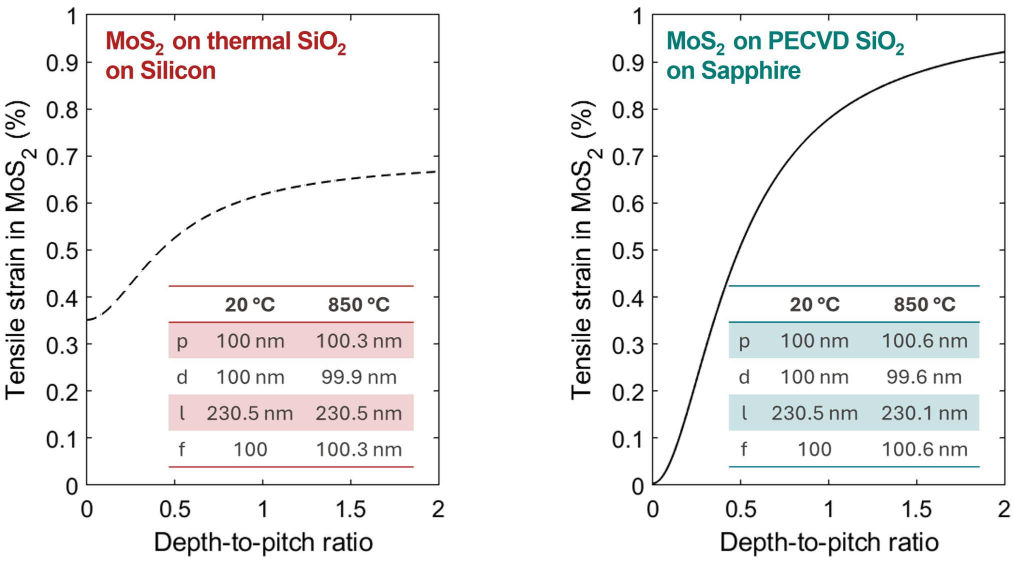

Figure S2: Comparison of induced strain in MoS_2 grown on (a) thermal SiO_2 on silicon and (b) PECVD SiO_2 on sapphire substrates.

In contrast to rounded, curved, inclined surfaces, materials grown on rectangular side profiles can only experience stress effects at vertical walls if the CTE mismatch and aspect ratio are high enough (resulting in a single strain value without controllable strain gradient), as the flat parallel surfaces do not contribute to the proposed strain-engineering approach. The surface contour length change in grayscale regions is the key in the presented work for strain formation. Thus, strain-induced bandgap engineering in 2D semiconductors in our work occurs across all patterned areas. When the CTE of the substrate exceeds that of the thin film, we observe fully tensile strain across all patterned areas.

The presented strain-engineered growth approach is not limited to high-temperature processes. Whereas the magnitude of induced strain would be slightly lower at lower growth temperatures due to the reduced thermal mismatch, BEOL integration is feasible in principle, and these strain levels will still be sufficient for effective

mobility enhancement in 2D-material transistors. For example, with a depth-to-pitch ratio of 0.5 at a temperature of 400 °C on a sapphire substrate, a strain of 0.25% is induced (see Figure S1). According to our previous work,[1] this would provide a mobility improvement of up to about 4x.

S2 Grayscale nanopatterning

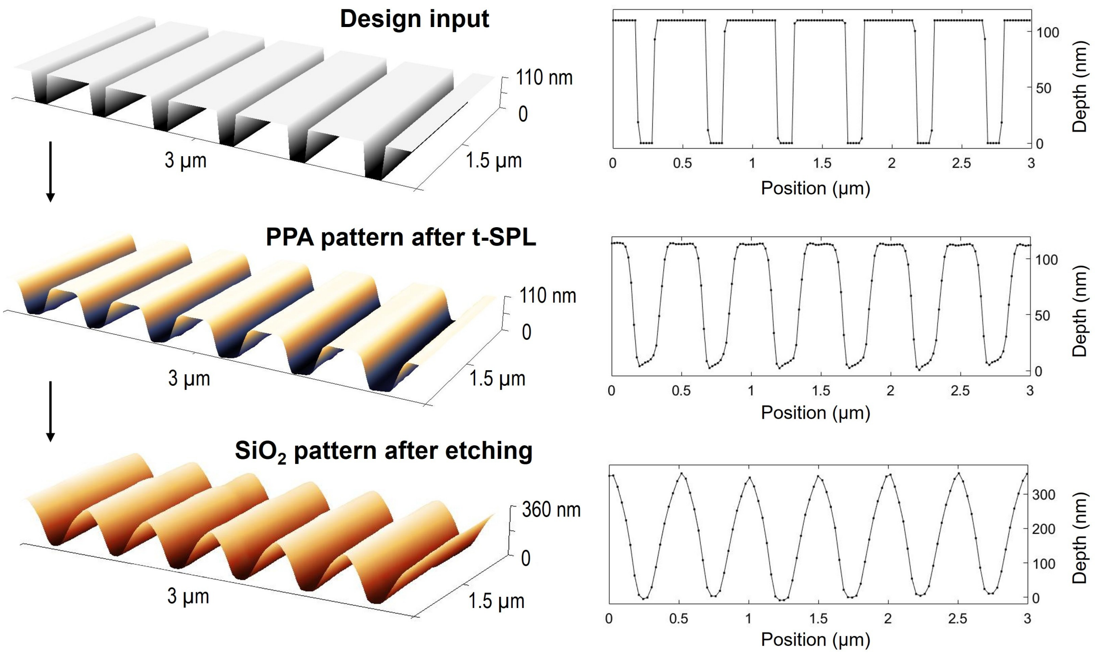

Figure S3: Strategy to obtain higher depth sinusoidal nanopatterns after t-SPL and plasma dry etching-based pattern transfer for low pitches (≤500 nm).

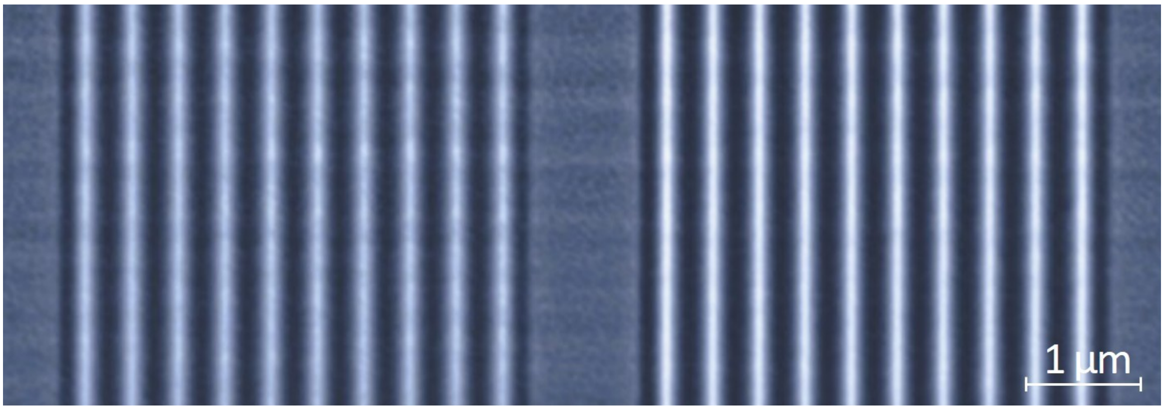

Figure S4: SEM image of sinusoidal nanopatterns used as a stamp for NIL.

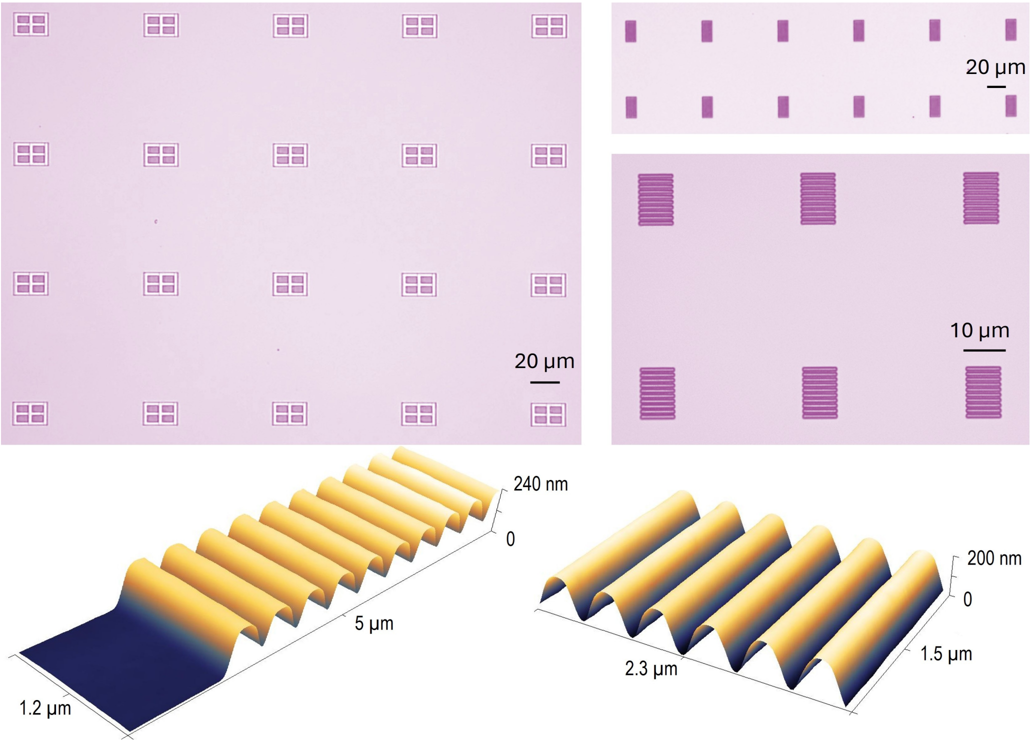

Figure S5: Optical microscope (top) and AFM images (bottom) of nanoreplicated polymer patterns on thermoplastic NIL resist.

RMS surface roughness change

	Reflow temperature			
Reflow time	90 °C	100 °C	110 °C	120 °C
1 min	-1%	0%		
2 min		0%	-13%	0%
3 min			-30%	
10 min			-22%	
15 min			-23%	
20 min			-29%	
30 min			-19%	
45 min				-31%
60 min			-13%	-25%
120 min			-36%	-33%
180 min			-45%	
1000 min			-36%	

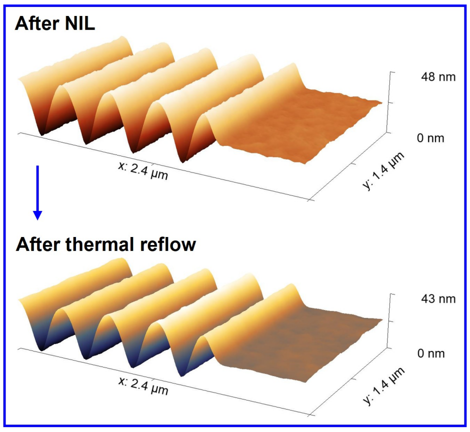

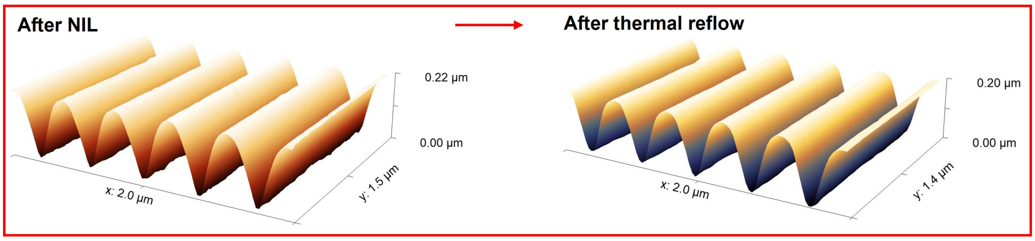

Figure S6: Thermal reflow experiments on imprinted NIL resist and the corresponding change in RMS surface roughness after heat treatments in the oven. Relatively high peak-to-peak changes were observed for thermal reflow at 120 °C.

S3 Challenges in transfer-based strain engineering

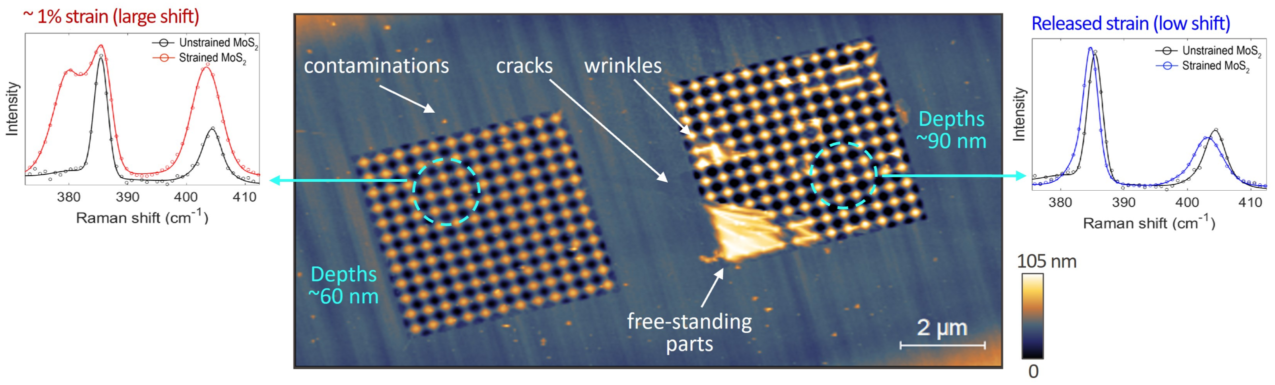

Figure S7: AFM image of MoS_2 monolayer grown on a sapphire substrate and transferred from the growth substrate to a pre-patterned target substrate through polymer-assisted imprinting, highlighting typical issues in transfer-based strain approaches. For low aspect ratio (0.13) grayscale nanopatterns, the 2D material conforms more uniformly to the surface, with reduced wrinkles, ruptures, and suspended regions. In contrast, all typical issues associated with material transfer are observed for the relatively higher aspect ratio (0.20). The 2D material conformally follows the lower–aspect-ratio sinusoidal surface (left) but fails to do so at slightly higher depths (right), and the strain is released if the material rips during transfer, as confirmed by Raman spectroscopy. Particularly, the nano- to micrometer scale cracks introduced during transfer can lead to failure of the final device. This image is adapted from the same work, which is based on a transfer-based strain approach, published as Erbas, B., et al. *Microsystems & Nanoengineering* 10.1 (2024): 28. [2]

S4 Strain engineered growth of 2D materials

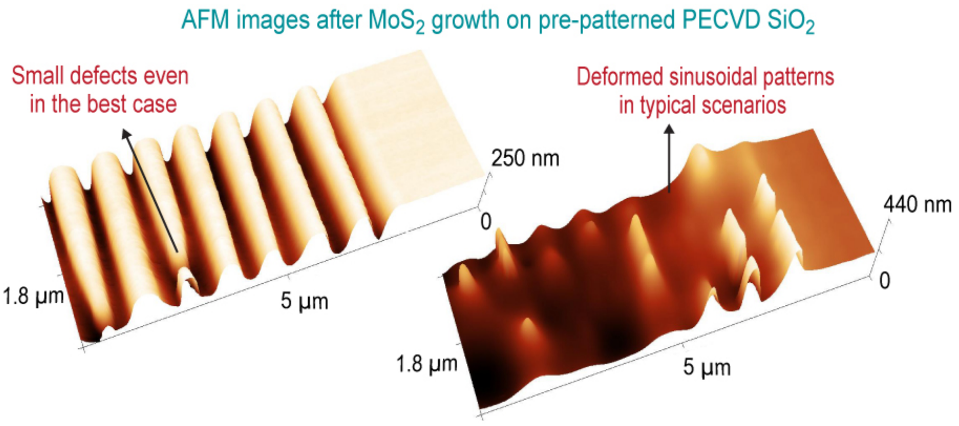

Figure S8: AFM images of MoS_2 grown on PECVD SiO_2 on sapphire substrates, showing successful MOCVD growth (left) and deformed surface profiles in typical cases (right).

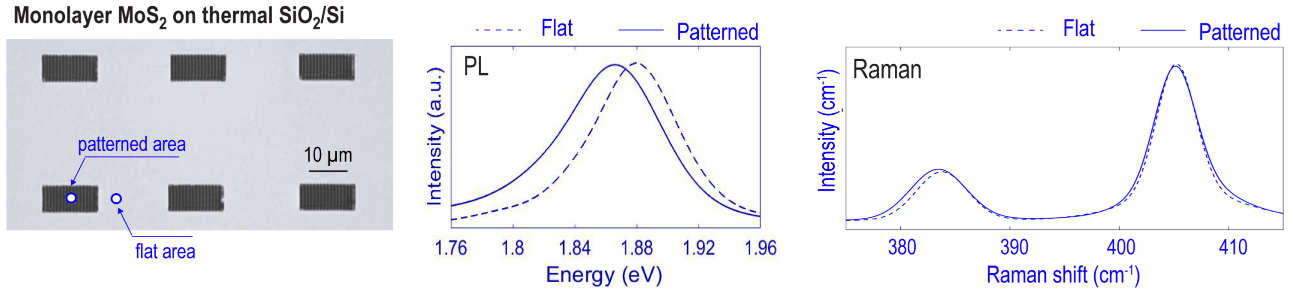

Figure S9: Strain characterization on flat and grayscale-patterned thermal SiO_2 surfaces with unidirectional sinusoidal profiles on a silicon substrate using PL and Raman spectroscopy, comparing spectral shifts within the same areas of interest. In addition to PL, Raman spectroscopy shows redshifts in the Raman peaks resulting from the induced tensile strain. For the Raman spectra, the intensity from the grayscale region is amplified by 3-fold. The E^1_{2g} Raman peak position shifts from 383.9 cm^{-1} to 383.3 cm^{-1} at a depth-to-pitch ratio of 0.33, corresponding to a calculated relative strain of 0.13%. This results in a redshift of 4.5 cm^{-1}/% strain, comparable to our previous works and the literature. [1-4]

In single-layer MoS_2, the intensity of the defect-activated LA(M) Raman mode at 227 cm^{-1} is widely recognized as a sensitive probe of structural defect density. [5] In the present study, no discernible LA(M) peak is observed for either MoS_2 grown on flat substrates or strained MoS_2 grown on the grayscale-patterned regions. The absence of this defect-related mode indicates a negligible concentration of structural defects in both cases, demonstrating that the strain engineering achieved through deterministic grayscale topographies does not introduce additional defect states into the MoS_2 lattice.

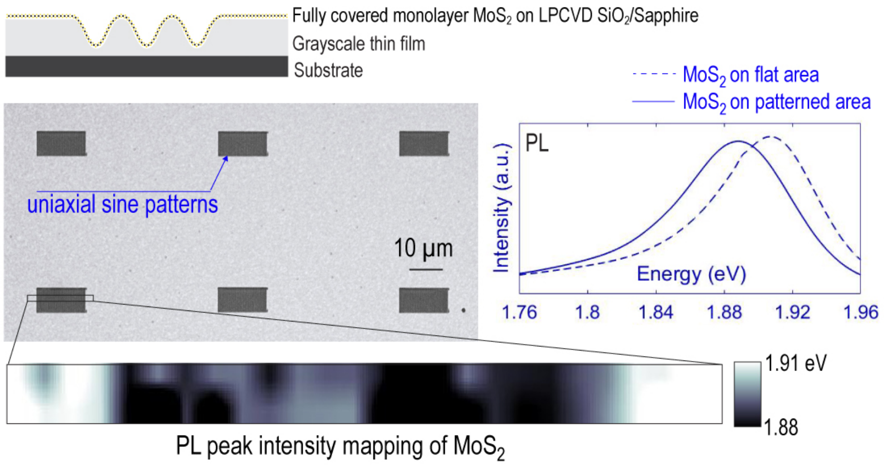

Figure S10: Strain characterization on LPCVD SiO_2 on a sapphire substrate patterned with unidirectional sinusoidal profiles.

In addition to micro-PL, we performed also TEPL measurements, as shown in Figure 4, to compare the quality of the grown 2D materials on various surfaces. Measurements from 55 points on thermal SiO_2 and 663 points on LPCVD SiO_2 show mean PL peak positions of 1878 meV with a standard deviation of only ±1.7 meV, and 1908 meV with a standard deviation of ±1.9 meV, respectively. These results support the quality comparison of MoS_2 grown on oxide layers fabricated by different techniques, confirming that heat treatments applied to densify the underlying LPCVD oxide results in 2D materials with PL characteristics comparable to those grown on untreated thermal oxide surfaces.

Table S1: Comparison of transfer-based strain in 2D materials with direct strained growth.

	Material growth & transfer-based strain	Transfer-free direct strained growth
**Process complexity**	Complex; detachment & alignment & transfer	Simpler; avoids material transfer
**CMOS integration**	Complex; less scalable and reproducible	Practical; highly scalable and reproducible
**Interface quality**	Unstable; lower quality; poor adhesion	Cleaner, stable with better adhesion
**Material quality**	Potentially higher quality, but transfer-limited	Potentially lower quality, but under development

The CTE mismatch between a substrate and a grown 2D material can induce strain, particularly notable tensile strain on fused silica substrates due to high CTE mismatch. However, this approach is limited because it relies primarily on the substrate's thermal properties rather than application-specific requirements, such as high thermal conductivity for high-performance processors where cooling is crucial or transparency across a broad spectrum for UV and IR applications. In contrast, our proposed method for strained growth of 2D material, benefiting from the CTE mismatch between a substrate and a 3D-patterned thin film, is calculated to be ~60% and ~164% more efficient for the MoS_2/3D-SiO_2/sapphire stack compared to the MoS_2/SiO_2 and MoS_2/Si stacks, respectively (for an aspect ratio of 2).

The presented strain approach proposes potential innovation in device architecture through 3D/grayscale surface topography engineering that introduces strain into the 2D semiconducting channels and improves their performance for future semiconductor scaling and innovation. This technique can be complemented by other efficient strain-engineering techniques such as thin-film stressor capping. Additionally, while stressor capping is a global method for inducing stress, grayscale nanopatterning enables local strain engineering and can be further tuned spatially through controlled variations in pattern amplitude and waviness.

Topographies with rounded, curved, or angled side profiles, specifically non-rectangular shapes such as sinusoidal, triangular, and trapezoidal patterns, exhibit significant surface length variations driven by thermal expansion and mechanical stress in the thin film. This differential behavior is especially advantageous and is unique to grayscale surface topographies, as it is not observed in rectangular-patterned surfaces, such as pillars, because their thin film surface remains parallel to the substrate surface. These proposed geometries differ from rectangular side profiles and are ideally periodic rather than randomly oriented. In the case of rectangular side profiles, such as pillar structures, only vertical sidewalls experience length variation; consequently, precise homogeneous strain control is not possible. This change in surface contour length is critical and cannot occur in rectangular-patterned surfaces, where the thin film surface is parallel to the substrate surface, as only vertical side profiles perpendicular to the surface plane can benefit from shrinkage in thin film thickness.

In addition to thermal properties (i.e., low CTE), another key reason for concentrating on SiO_2 layers is the potential for both deep and smooth grayscale patternability of these materials using lithography combined with dry plasma-based etch amplification, enabled by our developed and optimized recipes from earlier work and this study. However, additional layers can be deposited on top of these pre-patterned SiO_2 films, such as ALD-deposited Al_2O_3 or HfO_2, which are also advantageous for further surface smoothing, depending on application needs. In principle, the thin-film layer can be completely changed to an ideal dielectric depending on the final target application.

Table S2: Comparison of strain engineering techniques for 2D materials. The present work is highlighted in blue.

Techniques	Strain efficiency	Interface quality	Scalability	CMOS integration
**MEMS actuation**	High	Moderate; substrate-dependent	Very limited	-
**Indentation**	Locally High	Low; free-standing	Very limited	-
**Pressurization**	High	Low; free-standing	Very limited	-
**Heating**	Moderate	High; but surface-dependent	Limited	-
**Bending/stretching**	High	High; but surface-dependent	Very limited	-
**Thin film stressors**	Low to High	High; but surface-dependent	High	High; generic
**CTE mismatch**	Low to Moderate	High; but surface-dependent	High	Limited to substrate
**Crested prepatterns**	Moderate	Low; transfer-dependent	Moderate	Limited due to transfer
**Grayscale prepatterns**	Moderate to High	Moderate; transfer-dependent	Moderate	Limited due to transfer
**Direct strained growth**	Moderate to High	High; but surface-dependent	High	High; generic

Optical transparency at the device level could enable novel applications, particularly in the field of see-through/transparent electronics, which could significantly benefit from the integration of 2D material transistors, beyond the limits of conventional silicon-based technologies. For instance, 1 µm-thick SiO_2 on single-side-polished sapphire substrates exhibits transmittance greater than 11% in the 300–1000 nm wavelength range, as shown in Figure S11. When fully covered with a strained MoS_2 monolayer, the same substrate stack maintains transmittance above 9% in the visible range, with a maximum relative decrease of only ~4%. On double-side-polished sapphire wafers, which have transmittance exceeding 85%, the MoS_2/SiO_2/sapphire stack is expected to retain transmittance above 80%. Such transparent yet high-performance semiconductors are promising candidates for next-generation applications in displays, wearable electronics, optical sensors, and human–machine interfaces.

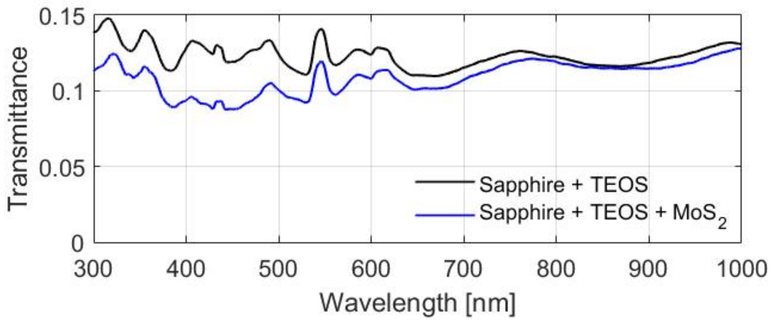

Figure S11: Transmittance comparison of 1 µm-thick SiO_2 on single side polished sapphire substrate with monolayer MoS_2 on 1 µm-thick SiO_2 on single side polished sapphire substrate.

References

[1] Liu, X., Erbas, B., Conde-Rubio, A. et al. Deterministic grayscale nanotopography to engineer mobilities in strained MoS_2 FETs. *Nat Commun* **15**, 6934 (2024).

[2] Erbas, B., Conde-Rubio, A., Liu, X. et al. Combining thermal scanning probe lithography and dry etching for grayscale nanopattern amplification. *Microsyst Nanoeng* **10**, 28 (2024).

[3] Li, H. et al. Optoelectronic crystal of artificial atoms in strain-textured molybdenum disulphide. *Nat. Commun.* **6**, 1–7 (2015).

[4] Conley, H. J. et al. Bandgap engineering of strained monolayer and bilayer MoS_2. *Nano Lett.* **13**, 3626–3630 (2013).

[5] Mignuzzi, S., Pollard, A. J., Bonini, N., Brennan, B., Gilmore, I. S., Pimenta, M. A., Richards, D. and Roy, D. Effect of disorder on Raman scattering of single-layer MoS_2. *Phys. Rev. B* **91**, 195411 (2015).
